# The Case for the Plaintiff

**Published:** 1913-03

**Authors:** 


### Department of Eugenics.

Conducted by Dr. Malone Duggan and Dr. Theo. Y. Hull.

### THE TEXAS STATE SOCIETY OF SOCIAL HYGIENE.

Headquarters: San Antonio, Texas.

DR. MALONE DUGGAN, President, San Antonio.
DR. F. E. DANIEL, 1st Vice President, Austin.
PROF. A. CASWELL ELLIS, 2d Vice President, Austin.
MRS. ELI HERTZBERG, 3d Vice President, San Antonio.
DR. THEO. Y. HULL, Secretary, San Antonio.
MR. W. L. EVANS, Treasurer, San Antonio.

EXECUTIVE COMMITTEE.

Dr. Malone Duggan, Chm.
Dr. Theo. Y. Hull, Sec'y.
Dr. W. F. McCaleb.
Hon. C. H. Jenkins.
Rev. A. W. S. Garden.
Hon. J. C. Willacy.
Mr. W. L. Evans.
Dr. Frank D. Boyd.
Dr. J. R. Nichols.
Dr. J. M. McCutchan.
Dr. W. A. King.
Dr. J. W. Torbett.
Dr. F. C. Walsh.
Dr. Joe Reuss.
Dr. Frank Paschal.

### The Case for the Plaintiff.

Marriage is the most important and sacred of human relationships. The stability of the State and society rests upon no other foundation. It means the establishment of the home—the family—in which practically every human interest centers. So important to human welfare and progress is the institution of marriage that society long ago placed around it certain restrictions. In the course of time, other conditions have arisen that call for still further regulation. With each recurring decade the knowledge is forced upon us that certain conditions now permissible, through the marriage relationship, spread disease, decrease human efficiency, and increase human degeneracy. It is, therefore, thought not only proper, but imperative, that remedial legislation should be attempted at this time. For this purpose, this measure, which seeks to establish a standard of physical fitness for marriage, is introduced.

When we ask that each applicant for a license to marry be required to submit competent evidence that he or she is physically fit, is not an alcoholic, an epileptic, an imbecile, a feeble-minded person, an habitual criminal, is not afflicted with advanced tuberculosis, and is not suffering with any active venereal disease, we do so because we know that these very conditions, each and every one of them, is detrimental to the race, increases human suffering, engenders crime, promotes the spread of disease and increases the burdens of the State. We, therefore, wish to submit the following evidence of the urgency of this measure:

Let us consider first the venereal diseases—gonorrhea and syphilis.

*Gonorrhea.*—It is said by competent authorities that 75 per cent of the adult male population suffer with gonorrhea some time during life, and that in this country over 500,000 young men of marriageable age contract the same disease annually. In the event of marriage with one of these, the healthy party will become infected in every instance. This entails upon the woman:

(a) Sterility—the childless marriages—50 per cent.

(b) Gynecological operations, 60 per cent.

(c) Inflammatory diseases, 80 per cent.

(d) Divorces, —? per cent.

It entails on the offspring:

(a) Of all blindness—25 to 40 per cent.

It entails on the State an unnecessary burden. It is estimated that every person blind from birth costs the State $10,000.

*Syphilis.*—Of all the blighting curses on human society, syphilis is probably by far the greatest. It leaves an unmistakable taint upon the succeeding generations. Neisser, a German authority, states that 15 per cent of the male population have this disease. Dr. Morrow, an American authority, places the number of syphilitics in the United States at 2,000,000.

In the event of marriage with the syphilitic, the healthy party will be infected in every instance. It causes:

(a) In women—42 per cent of all abortions, and may be followed by apoplexy, locomotor ataxia, general paresis or insanity.

(b) In the offspring—60 per cent of all stillbirths; about 15

per cent of our high infant mortality, and is responsible for a large per cent of feeble-mindedness, epilepsy, idiocy, insanity and criminality.

(c) And entails on the State a great burden for the support of the defectives, paupers, insane and criminals.

*Tuberculosis.*—The universality of this disease is a matter of common knowledge. Probably 50 per cent of all living human beings have it in either the latent, healed or active form. In England, 24.6 per cent of the children of non-tuberculous parents, and 33.15 per cent of the tuberculous parents contract the disease (Squires). Dr. Sachs, of Chicago, found among the laboring people that 53.1 per cent of the living children of tuberculous parents were infected.

In the event of marriage with one afflicted with active pulmonary tuberculosis, the healthy party runs a grave risk of infection (at least 25 per cent). The children of such are invariably weak, and at least 35 per cent contract the disease. While it is difficult to place a monetary value on a human life, all authorities agree that the loss to the State is enormous.

*Alcoholism and Epilepsy.*—To those familiar with these conditions and the effect of marriage among such, the granting of a marriage license to either an alcoholic or an epileptic seems nothing less than an absolute crime. It fails to consider the rights of the unborn. The offspring of such may be feeble-minded, epileptic and idiotic, and may become criminals or insane.

*Heredity.*—Let us view, briefly, marriage among the physically unfit from the standpoint of heredity. It is known that 60 per cent of the insane give a family history of insanity, epilepsy, or kindred disorders. We know that congenital defects are largely due to parental defects. We also know that the physical, mental and moral characteristics of the parents are inherited by the offspring.

The following family histories are suggested:

*Family No. 1.*—Descendants of woman addicted to alcoholism. Number of offspring, 709. Among these were 101 illegitimate children, 181 prostitutes, 142 beggars, 46 workhouse inmates, 76 criminals, and many more or less habitual drunkards. The cost of this family to the State was $1,200,000.

*Family No. 2.*—This represents the progeny of a feeble-minded man, the illegitimate offspring of a normal man and a feeble-minded woman. Number of offspring, 480. Of these, 36 were illegitimate, 33 sexually immoral, 24 confirmed alcoholics, 3 epileptics, 82 died in infancy, 2 criminals, 8 keepers of houses of ill-fame, and 143 were distinctly feeble-minded.

*Degeneracy.*—The following evidence of degeneracy found within institutions of the United States is submitted: Insane and feeble-minded, 300,000; blind, 100,000; deaf and dumb, 100,-000; prisoners, 100,000; juvenile delinquents, 23,000; paupers, 100,000, and other persons cared for by charity 2,000,000, which is an increase of 125 per cent in fifty years. (This does not include those outside of institutions.)

COMPARATIVE COST TO THE PUBLIC.

| | |
|---|---|
| Immorality and social disease | $3,000,000,000 |
| Liquor | 2,000,000,000 |
| Tobacco | 1,200,000,000 |
| Jewelry and plate | 800,000,000 |
| Automobiles | 500,000,000 |
| Church work at home | 250,000,000 |
| Confectionery | 200,000,000 |
| Soft drinks | 120,000,000 |
| Tea and coffee | 100,000,000 |
| Millinery | 90,000,000 |
| Patent medicines | 80,000,000 |
| Chewing gum | 13,000,000 |
| Foreign missions | 12,000,000 |

